# Experience of Respite Care with Alternating Housing Among People with Dementia: A Mixed-Methods Study

**DOI:** 10.3390/geriatrics11040110

**Published:** 2026-08-19

**Authors:** Mirkka Söderman, Annelie K. Gusdal, Lena-Karin Gustafsson

**Affiliations:** Department of Health Sciences, Innovation and Design, Mälardalen University, P.O. Box 325, 631 05 Eskilstuna, Sweden; annelie.gusdal@mdu.se (A.K.G.); lena-karin.gustafsson@mdu.se (L.-K.G.)

**Keywords:** critical incident technique, dementia, family care, informal caregivers, interviews, municipal care, respite care

## Abstract

**Background**: In line with international aging policies, most people with dementia receive care at home. Community-based respite services aim to relieve, support, or share caregiving responsibilities. More person-centered knowledge based on experiences from this group is needed to increase informed decisions about planning of qualitative respite care. The aim of this study is to describe how people with dementia experience respite care with alternating housing, i.e., living partly at home, partly in residential care, refer to their everyday life and well-being. **Methods**: A mixed-methods design was used. The qualitative approach focused on variations of dilemmas in interviews with people with dementia who were granted and received respite care (n = 8). Also, a quantitative approach focused on quality of life using behavioral and psychological symptom scores. **Results**: Everyday life at the respite care accommodation described by people with dementia included morning routines, variation in the environment, activities in the company of roommates, but also a lack of activities. Social belonging was described by people with dementia through living with roommates and through social contact with the healthcare staff. Quality of life and behavioral and psychological ratings varied between individuals and settings, but no consistent pattern was identified. **Conclusions**: Respite care appears to maintain a high quality of life and a low level of psychological symptoms for most of the people with dementia. Although the sample was small, these results are nevertheless an important contribution to the phenomenon, experiences of respite care by people with dementia, that need further research. Furthermore, these results can give decision-makers guidance for professional care efforts.

## 1. Introduction

In line with international aging policies, most people with a dementia diagnosis receive care in their own homes on a long-term basis [1]. As most older people prefer to age in their own home, they remain there as their care needs intensify [2] and should be given good conditions for doing just that. To age in place means living with preserved independence, having influence over everyday life and opportunities for an active life [3], and enjoying independent living with safe conditions and a meaningful existence with others [4]. However, improved life expectancy has led to increased years of life spent with morbidities, such as dementia, which increases support needs [5].

In this study, we focused on informal care and how this can be shown in the light of older persons’ needs [6]. Informal care provided by families constitutes a major part of the care older persons with dementia receive [7]. The foundation of person-centered care is respect for the person, the person’s right to self-determination, and equal understanding, and the complexities of care in the home often include relational and social aspects. The older adult, as well as the informal caregiver, is expected to be an active partner in their own care but is often excluded from the information flow [8].

For persons who are in the initial stages of dementia, depending on their family situations, there is often a spouse, child or children, or an informal or family caregiver who takes responsibility for the care of the person with dementia [9]. This increases the demands on family members who must care for their loved ones around the clock. Furthermore, a large number of people with dementia pose major challenges not just for their families but also for social care, as well as social services. However, as the disease progresses into its advanced stages, care provided by professional caregivers is necessary. Involved caregivers must address the needs of the entire family to prevent extended health problems and stress for everyone involved [10]. At the same time, Pollock et al. [9] highlight the need to recognize the family and their informal care as functioning care units of crucial importance for people with dementia.

There are a range of community-based healthcare services available to people with dementia living at home, including home-based interventions and/or respite care outside the home [11]. Respite care in particular aims to give family caregivers time to rest and recover from their emotionally and physically demanding care responsibilities, as it provides family caregivers with opportunities for respite, both on a regular basis and in unexpected or emergency situations. There are several forms of respite care, such as special residences, respite care at home, respite care with alternating housing, and day care, and therefore, the design can differ both in frequency and execution [12,13]. According to O’Shea et al. [14], respite services have traditionally been seen as services from the perspectives of family caregivers, while the perspectives of people with dementia have been largely ignored. However, it appears that people with dementia can have difficulty accepting respite care; some express that the environment is experienced as cold, that there is a lack of commitment, and that they prefer to be cared for at home instead [15]. Furthermore, enjoyable daily activities increase people with dementia’s self-worth, happiness, and energy levels [16] and can be accepted to a greater extent than respite care with alternating housing [15]. Research also shows that people with dementia living in ordinary housing have significantly higher QoL than those living in residential care [17]. Earlier research has mainly examined respite care from the perspectives of family caregivers and service providers or has studied respite services as a broad category. Limited evidence is available about how people with dementia themselves experience the specific arrangement of alternating between their ordinary home and an out-of-home respite care residence, a common form of care in Nordic countries. It is also unclear how their accounts of everyday life in alternating housing relate to indicators of quality of life and behavioral and psychological symptoms across the two settings.

The municipal care and social welfare administration needs to make considered health-promoting decisions that suit both people with dementia and their families. Therefore, the aim of this study is to describe how people with dementia experience respite care with alternating housing and how such care relates to their everyday life and well-being.

## 2. Materials and Methods

This study used a convergent mixed-methods design with qualitative priority. Qualitative data from interviews conducted according to the critical incident technique (CIT) and quantitative data obtained using validated instruments were collected during the same study period and from the same participants. The two strands were analyzed separately and subsequently brought together during the interpretation of the findings. The qualitative strand was given priority because the primary focus of the study was the participants’ experiences of respite care, whereas the quantitative data were used descriptively to complement and contextualize the qualitative findings. The study was conducted according to the research formulation, research planning, and research implementation stages described by Leech and Onwuegbuzie [18].

### 2.1. Participants and Recruitment Procedure

The study was created and developed in collaboration with a medium-sized municipality’s care and social welfare administration, and they also supported the selection process of participants. All people who received respite care with alternating housing due to dementia in the municipality during this period (n = 16) were invited to this study. However, people in very late stages of dementia who were unable to give informed consent or respond verbally to the semi-structured questions were excluded. Capacity to provide informed consent was assessed by the research assistant before inclusion and was reassessed at the data collection visit. The assessment was specific to the decision about research participation and focused on whether the person could understand, in broad terms, the purpose of the study, what participation involved, that participation was voluntary, and that they could decline or withdraw without consequences. The person also needed to be able to communicate a consistent choice regarding participation. Information was provided verbally and in writing and was adapted to the participant’s cognitive and communicative abilities.

A list of people with dementia who had been granted respite care with alternating housing was provided to the research team by the municipality’s care and social welfare administration. However, information on specific dementia diagnoses among the participants was not provided. One week after sending an information letter regarding the research study, the people with dementia were contacted by telephone, informed about the study, and asked about participation. If the person consented, a visit was booked for data collection. In total, nine persons with dementia consented to participate and were included in the study, although one person was excluded at a later stage due to an inability to answer interview questions due to more severe cognitive impairment than expected at inclusion. A request for participation was sent in August 2022, with data collection from September 2022 until January 2023. The participants also received a verbal reminder of the content of the missive letter before the interview and measurement with validated instruments began. They were also informed that they could withdraw their participation at any time.

### 2.2. Context

Like other Nordic countries, Sweden has developed a tax-funded elderly care system administered by the National Board of Health and Welfare and the municipalities [4]. The services are regulated by the Social Services Act, with responsibility at the municipal level. The design differs from that of Southern and Central Europe, where family responsibilities and informal care carry more weight than public institutions.

All included participants were granted respite care at the same residence; housing was shared both by people with dementia and those without a dementia diagnosis. The residence was on two floors with seven rooms per floor. A daycare center was located on the ground floor of the same building, although the people with dementia receiving respite care rarely used this facility. In total, about 30 participants received care on a rotating basis, some of them for two weeks at a time, others for one week. Healthcare staff tried to arrange, if possible, for people with dementia to have the same room on each occasion. In their “own” rooms, they had a bed and bedside table, a large TV on the wall, and a table with two armchairs and a few paintings. Each room had its own toilet and shower. On each floor, there was also a smaller living room as well as a kitchen with dining areas. Outside the residence, there was a courtyard.

### 2.3. Data Collection

Consent was treated as an ongoing process throughout the visit. Before data collection began, the interviewer repeated the study information and verbally confirmed that the participant remained willing to take part. During the interview and assessments, the interviewer monitored both verbal and non-verbal indications of willingness, discomfort, fatigue, distress, or withdrawal. Participants were offered pauses and were reminded that they could decline answering individual questions or end the visit. If a participant appeared distressed, repeatedly disengaged, or indicated verbally or non-verbally that they did not wish to continue, data collection was paused or terminated. In one case, the interview was discontinued because the participant’s cognitive impairment made it impossible to establish continued understanding and willingness to participate. Data collection took place both in the ordinary housing and in the respite care residence and was carried out by research assistants working on the project, with the support of the family caregivers at the ordinary housing and healthcare staff at the respite care residence who were familiar with the people with dementia.

Data were collected via individual semi-structured interviews and measurements with validated instruments. The interviews were conducted according to the CIT, which encourages reporting on dilemmas and consequences. People with dementia focused on their daily lives both in their ordinary home and in the respite care residence and were given the opportunity to describe both positive and negative critical incidents they perceived as important [19]. The interviews followed a semi-structured interview guide (Appendix 1). Quality of life was measured via the Swedish version of the Quality of Life in Late-Stage Dementia (QUALID) scale [20], shown to work well in community settings [21]. The QUALID scale consists of the following behaviors: smiling, sadness, crying, emotional discomfort, physical discomfort, dissatisfaction, irritability/aggression, appetite, enjoying physical contact, enjoying social contact, and appearing calm and relaxed. The statements are ranked on a five-point scale, where lower scores represent a higher quality of life. Behavioral and psychological symptoms in dementia (BPSD) were assessed with the Swedish version of the Modified Neuropsychiatric Inventory Scale—Nursing Home (NPI-NH) [22], which works well in community settings [23,24]. The NPI-NH scale consists of the following Behavioral and Psychological Symptoms of Dementia: delusions, hallucinations, agitation, depression, anxiety, euphoria, apathy, disinhibition, irritability, abnormal spatial/motor behaviors (restlessness and wandering), nocturnal behavior (sleep), and appetite and eating disorders. For each of these, occurrence, frequency, and severity are assessed to obtain a summed symptom score of the total NPI score.

All participants were interviewed once. Measurement with instruments was performed once both in ordinary housing and in the respite care residence.

At data collection sessions conducted in the participants’ ordinary homes, a family caregiver could be present to provide reassurance and communication support. The interviewer addressed the questions directly to the person with dementia and allowed sufficient time for the participant to respond. Family caregivers could help by repeating or clarifying a question when needed, but they were asked not to answer on the participant’s behalf. Statements made by family caregivers were not treated as qualitative interview data or included in the CIT analysis. Their supportive presence did not replace the participant’s own consent or expressed willingness to participate.

The QUALID and NPI-NH instruments were administered by the same member of the research team on both measurement occasions. Because the instruments require observations of the participant’s behavior over time, the ratings were based on information provided by a person familiar with the participant in each setting. For the assessment in the participant’s ordinary home, the family caregiver who was involved in the participant’s daily care provided the observational information. For the assessment at the respite care residence, the information was provided by a member of the healthcare staff who was familiar with the participant during the respite care period. The researcher reviewed the instrument items with the respective informants and recorded the ratings.

Thus, the same researcher administered the instruments in both settings, but the proxy informant differed between settings. Family caregivers provided the information for ratings in the ordinary home, whereas healthcare staff provided the information for ratings at the respite care residence. Each participant was assessed once in each setting.

### 2.4. Analysis

The qualitative data were analyzed and categorized according to the Critical Incident Technique described by Flanagan [19], and further developed for nursing and healthcare research by Fridlund et al. [25], with a focus on the variation of dilemmas in the context of respite care. The interviews were transcribed verbatim, and to create a first understanding of the data, an initial listening and reading of the interviews while taking notes was carried out, followed by repeated comprehensive reading of the interviews to become familiar with the content. A total of 140 critical incidents were identified from the transcripts. Any description of an incident in terms of dilemma or consequence was considered a unit for further analysis. The incidents with perceived dilemmas were delimited and placed in a structural analysis table by dividing the meaning units into subcategories. Categorization was based on representing the general nature of the subcategories that described the main areas. The initial delimitation of transcripts—identifying positive and negative events—was carried out by one of the researchers, whereas the categorization of meaning units was performed jointly, with any disagreements discussed.

The qualitative and quantitative data were initially analyzed separately. Integration occurred at the interpretation stage, when the findings from the QUALID and NPI-NH assessments were considered alongside the qualitative categories describing the participants’ experiences of daily activities and social togetherness. Because of the small sample, the quantitative findings were used descriptively to complement and contextualize the qualitative findings rather than to test differences between the two housing settings.

The analytical framework was validated, and credibility was achieved through discussions throughout the analysis process between the researchers in the project group who represent different professional experiences as well as different academic levels. Descriptive statistics regarding data from the validated instruments were conducted via Excel.

### 2.5. Ethics

This project was approved by the Ethics Committee in Lund (protocol code 2022-01835-01 and “19 April 2022”). Moreover, the study was carried out in accordance with the World Medical Association Declaration of Helsinki [26] and informed consent from all participants and their involved family caregivers was obtained in accordance with the Swedish Data Protection Authority (DPA) GDPR (2016/679). Further, the invited participants received information that participation was voluntary and that the data collected would be handled in such a way as to ensure that only authorized persons have access.

## 3. Results

### 3.1. Background Information

The mean age among the participants in this study was 81.6 years, with one female and seven males. All males were married, and the female was a widow. Five of the participants lived in their own villa, and three lived in apartments.

### 3.2. Results from Measures with Validated Instruments

Overall, the quality of life for the participants measured with the QUALID-instrument was scored as good, i.e., a mean of 19.5 points at home and a mean of 22.8 points in respite care, except for one person scoring 13 points at home and 15 points in respite care (Table 1). The behavioral and psychological symptoms scores measured with the NPI-NH instrument varied to some extent according to housing situation (ordinary housing vs. respite care residence); however, even if there were variations between the estimates among the people with dementia, there was no clear pattern as to how the alternating living situation affected them. In some cases, behavioral symptoms occur at home but not at the respite care residence, or vice versa.

### 3.3. Results from Analyzed Interviews

**Figure 1:**
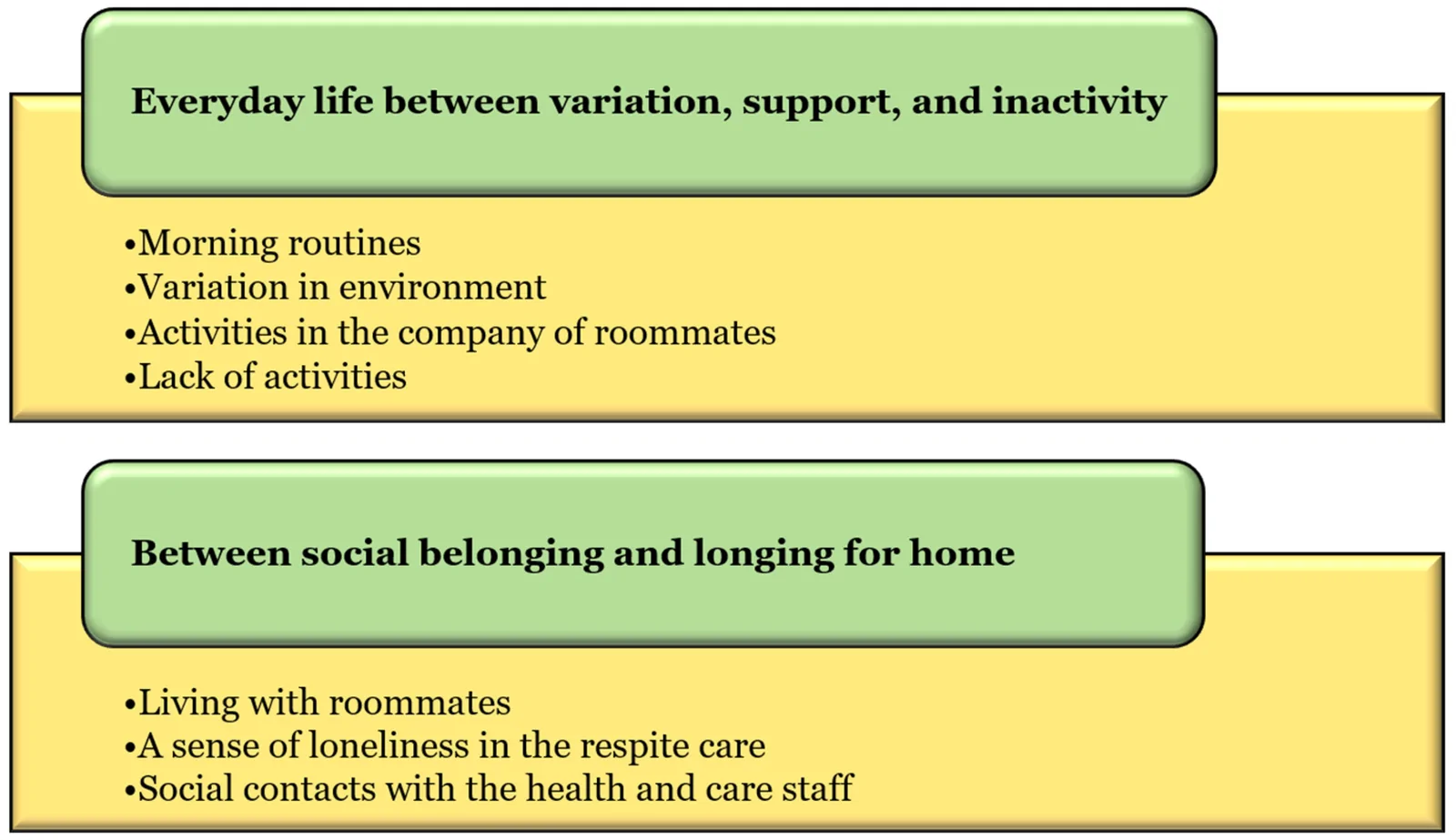
Summary of categories and subcategories of experiences of respite care among people with dementia.

The participants’ accounts indicated that alternating housing could be experienced both as an enriching change in everyday life and as a disruption associated with inactivity, loneliness, and longing for home. Opportunities for support, meaningful activities, social interaction, and contact with familiar staff contributed to positive experiences and appeared to support well-being. In contrast, when activities and social contact were limited, the respite care stay could be experienced as passive and isolating. The variation in the participants’ experiences was captured in two categories: Everyday life between variation, support, and inactivity and between social belonging and longing for home (Figure 1). These categories answered the following part of the aim: to describe how respite care is experienced by people with dementia. In the presentation of the categories, italicized text refers to the subcategories.

#### 3.3.1. Category 1: Everyday Life Between Variation, Support, and Inactivity

Alternating housing referred to everyday life by providing assistance with daily routines, a change of environment, and opportunities to participate in activities. However, the participants’ experiences varied depending on the support and activities available during their stay.

The participants described needing assistance with *morning routines*, particularly getting out of bed and dressing. Such support was provided by family caregivers in the ordinary home and by healthcare staff at the respite care residence.

The participants described *morning routines* as an important part of everyday life. The participants woke up in the morning, but when there was no help to get out of bed and their legs were too weak, it could cause a fall, leading to a fracture. They needed the support of a relative in the ordinary housing or healthcare staff at the respite care to get dressed.

“Yes, you try … If there are two of us, then there are no problems. But it gets too much, so to speak, just when you must get dressed and things like that. You must put on a shirt sleeve and stuff, that’s what’s tricky. My wife is the one who handles that”.

(Respondent 5)

These descriptions indicate that continuity in everyday routines depends on access to another person who could provide practical assistance. Alternating housing did not remove the need for support but transferred responsibility for this support from the family caregiver at home to the healthcare staff during the respite care stay. Activities at the respite care were also described by the participants as *variation in environment*. Even indoors, they could walk back and forth a bit, stopping and talking to someone. However, the opportunity for walks and outdoor stay was especially appreciated.

“I think this is great because sometimes you must go out and do things like this. I think that’s important, because I feel that you feel so damn locked in when you are inside the walls. And getting outside…well, I have always liked being outdoors”.

(Respondent 1)

For these participants, the change of environment represented more than moving between two housing settings. It created opportunities to move around, meet other people, and engage in activities outside the private room, thereby adding variation to everyday life.

The change of environment was appreciated by the participants as they had the opportunity to perform *activities in the company of roommates*. This could be some entertainment arranged by the healthcare staff at the respite care, playing board games or cards together, having a question-and-answer session, singing time, or small chats and meals in the communal area.

“Yes, when you are there, yes. They often have something, maybe a question-and-answer session or that we do something more practical, like that. And you are part of it. And then you might even have a singing session [laughs] sometimes”.

(Respondent 6)

Participation in shared activities appeared to make the respite care stay more meaningful by providing structure, stimulation, and opportunities to be involved with others. The participants’ experiences therefore depended not only on being offered respite care but also on what they were able to do during the stay.

However, some of the participants stated that there was a *lack of activities* and that there was nothing special on the days at the exchange of accommodation except for watching TV, news, or sports: “Well, then I don’t do much. Looking around there” (Respondent 2).

These accounts demonstrate an important variation in the findings. While alternating housing could enrich everyday life through environmental change and shared activities, it could also be characterized by inactivity when meaningful activities were unavailable. Thus, the significance of alternating housing for everyday life was closely related to the extent to which participants received practical support and had opportunities for individual or shared engagement during their stay.

#### 3.3.2. Category 2: Between Social Belonging and Longing for Home

The participants’ accounts showed that social relationships were central to how they experienced everyday life and well-being during alternating housing. Contact with other residents and familiar healthcare staff could create companionship, safety, and a sense of belonging. However, when opportunities for social interaction were limited, the respite care stay could instead be associated with loneliness, disconnection, and longing for home.

Social contacts at the respite care were described by the people with dementia partly through *living with roommates*. They meant that the most enjoyable part of the respite care was having friends. Even if they did not know each other before, they were all in the same situation and age. The participants described that they hung out with each other and had their daily meals together in the common area; they talked to each other and participated in various activities at the respite care together: “… we got along very well … I think everyone was enjoying the company of each other” (Respondent 9).

For these participants, being together with other residents made the respite care residence more than a place where practical care was provided. Shared meals, conversations, and activities created opportunities for participation and contributed to a sense of social belonging during the stay.

In contrast, some of the participants stated a sense of *loneliness in the respite care*. They sat alone, mostly watched TV without much contact with others and longed to go home: “Missing home… I would like to be at home like before” (Respondent 7).

These accounts highlight that alternating housing did not provide companionship or a sense of belonging for everyone. For some participants, limited social interaction and separation from the familiar home environment made the stay feel isolating. Longing for home therefore represented an important contrasting experience to the accounts of companionship and enjoyment described by other participants.

Other social contacts at the respite care were *the social contacts with the healthcare staff*.

“Well, there I am talking to her. She is so very nice… Yes, because she is a very nice head nurse. You get in touch with her directly. The staff are also … ordinary people. Kindness. And very kind to the neighbors…”.

(Respondent 8)

Positive encounters with staff appeared to contribute to the participants’ sense of being acknowledged and cared for as individuals. Staff members’ availability, kindness, and willingness to engage socially were therefore important not only for the provision of practical assistance but also for participants’ social well-being during the respite care stay.

Encounters with the healthcare staff gave a sense of familiarity among people with dementia. The participants described that healthcare staff talked to them, helped when needed, and participated in activities. A stable healthcare staff group, mostly consisting of the same people, gave a sense of safety and security among the people with dementia. Substitute or replacement of healthcare staff could be perceived as being unsuitable, and there could often be a staff shortage. However, those who were perceived as less suitable were handled by the regular healthcare staff in a way that did not cause inconvenience to the participants.

These findings indicate that continuity among staff helped make the respite care environment more familiar and predictable. Relationships with regular staff could support feelings of safety and belonging, whereas staff shortages and unfamiliar personnel risked weakening this continuity. Thus, participants’ experiences of social togetherness depended not only on contact with other residents but also on the availability, continuity, and interpersonal approach of the healthcare staff.

Taken together, alternating housing could support well-being by offering companionship, social participation, and trusting relationships with familiar staff. However, these benefits were not experienced uniformly. When contact with others was limited or the respite care environment did not provide a sufficient sense of familiarity and belonging, participants could experience loneliness and a strong desire to return home.

### 3.4. Synthesis

Although the qualitative accounts revealed both positive experiences and experiences of inactivity, loneliness, and longing for home, these variations were not reflected in a consistent pattern in the QUALID or NPI-NH ratings across the two settings. The instrument data therefore complemented the interviews by indicating that the meaning of alternating housing varied between individuals rather than producing a uniform change in quality of life or behavioral and psychological symptoms.

## 4. Discussion

This study contributes novel insights into how people with dementia experience respite care in the form of alternating housing, a care arrangement that is relatively underexplored from their own perspective. By integrating qualitative data from interviews using the CIT with quantitative measures of QoL and BPSD, the study offers a qualitatively driven mixed-methods perspective. The findings show that alternating housing was not experienced uniformly. It could provide practical support, variation in everyday life, meaningful activities, companionship, and relationships with familiar staff, but it could also be associated with inactivity, loneliness, insufficient belonging, and longing for home. The significance of alternating housing for everyday life and well-being therefore appeared to depend on how the care environment responded to the individual participant’s needs, preferences, and opportunities for engagement.

The QUALID and NPI-NH assessments varied between individuals and, in some cases, between the ordinary home and the respite care residence. However, there was no consistent pattern indicating that either setting was uniformly associated with better quality of life or fewer behavioral and psychological symptoms. The findings should therefore not be interpreted as demonstrating that alternating housing maintained or improved quality of life or reduced BPSD. Rather, the relatively favorable QUALID scores for several participants, together with the absence of a uniform pattern in NPI-NH symptoms, suggest substantial individual variation. Previous research has reported that people with dementia living at home have a significantly higher quality of life than those living in respite care [17,27]. The present study adds a more nuanced perspective by examining an arrangement in which participants continued to live at home but regularly stayed in a respite care residence.

The integration of the qualitative and quantitative findings is important in interpreting these results. Although the interviews revealed both positive experiences and experiences of inactivity, loneliness, and longing for home, these differences were not reflected in a consistent direction in the QUALID or NPI-NH ratings. One possible interpretation is that the meaning of alternating housing was highly individual and situational. Standardized proxy-based assessments may identify observable behaviors and symptoms, but they may be less sensitive to experiences such as meaningful engagement, familiarity, belonging, or a desire to return home. The qualitative findings therefore added dimensions of everyday life and subjective meaning that were not fully visible in the instrument scores. At the same time, the absence of a consistent quantitative pattern cautions against assuming that positive or negative interview accounts represented a general effect of the respite care arrangement.

The first qualitative category, *Everyday life between variation, support, and inactivity*, showed that alternating housing could refer to the organization and content of everyday life in several ways. Participants needed practical assistance with morning routines and dressing in both settings. Alternating housing did not remove this need but temporarily transferred responsibility for providing support from family caregivers in the ordinary home to healthcare staff at the respite care residence. This transfer of responsibility is central to the function of respite care, but the participants’ accounts also showed that the quality of the stay depended on more than the provision of basic assistance. Opportunities to move around, spend time outdoors, participate in shared activities, and experience a change of environment could add structure, stimulation, and variation to everyday life. Moreover, the findings support Moore’s [5] findings that quality of care is more important than care setting per se, as the built environment, staff continuity, and activity programming strongly influence subjective well-being.

These findings are consistent with previous research showing that physical environments and communal spaces that facilitate engagement in a variety of activities are associated with higher quality of life among people with dementia [28]. Participation in activities is also an important aspect of well-being among people at different stages of dementia [29] and shows that meaningful activity may enhance self-worth, happiness, and energy levels [16]. However, the present findings also demonstrate that the presence of an activity program or communal environment does not necessarily result in meaningful engagement for every individual. Some participants described having little to do apart from watching television. For them, the change of setting did not necessarily enrich everyday life but could instead involve passivity and a lack of meaningful content. The findings therefore emphasize the difference between activities merely being available and activities being accessible, personally meaningful, and adapted to the individual’s interests and abilities.

The second category, *Between social belonging and longing for home*, showed that social relationships were central to participants’ experiences of respite care. Shared meals, conversations, and activities with other residents could contribute to companionship and a sense of belonging. For some participants, being with people of a similar age and in comparable circumstances was one of the most valued aspects of the stay. Social contact therefore appeared to make the respite care residence more than a setting in which practical care was delivered. It could become a place in which participants were included in a social community.

However, this experience was not shared by all participants. Some spent considerable time alone, had limited contact with others, and expressed a strong desire to return home. These accounts need to be understood as a central variation in the findings rather than isolated negative experiences. Longing for home may reflect attachment to familiar routines, relationships, surroundings, and identity, but it may also indicate that the respite care environment did not provide sufficient social connection or meaningful engagement for that person. Previous research has similarly shown that access to and acceptance of respite services is influenced by how well the service is perceived to correspond to the needs of people with dementia and their family caregivers [15]. Studies focusing specifically on people with dementia have also identified an implementation gap between the principles of person-centered care and how some respite services are experienced in practice [14]. The findings suggest that reluctance to remain in respite care should not automatically be interpreted as resistance caused by dementia. It may communicate an unmet need for familiarity, belonging, activity, or greater individual adaptation.

The role of healthcare staff also emerged as central to participants’ experiences. Positive interactions with staff who were kind, available, and willing to talk contributed to participants feeling acknowledged and cared for as individuals. Regular staff members created familiarity, predictability, and safety, whereas temporary staff and staff shortages risked weakening continuity. These findings are consistent with person-centered approaches to dementia care, which are built around the needs of the individual and depend on knowing the person through an interpersonal relationship [30]. Staff continuity may be especially important in alternating housing because participants repeatedly move between two environments. Familiar staff can help bridge these transitions by recognizing the individual’s routines, preferences, communication patterns, and need for reassurance.

Taken together, the two qualitative categories indicate that the quality of alternating housing cannot be assessed solely by whether a place, room, or period of respite is provided. What appears to matter is how everyday life is constituted during the stay. Practical support, meaningful activity, opportunities for social participation, and familiar relationships could support well-being. Conversely, inactivity, limited interaction, unfamiliarity, and insufficient individual adaptation could make the same form of care isolating. Alternating housing should therefore be understood as a relational and contextual care arrangement whose meaning is shaped by what happens within each setting and during the repeated transitions between them.

Alternating housing also presupposes that substantial informal care is provided during the periods when the person with dementia is living at home, most commonly by a spouse or another family member. Previous research indicates that the use of out-of-home respite services is associated with circumstances relating to both the person with dementia and the family caregiver, including the caregiver’s perceived caregiving demands and the duration of caregiving [11]. The interdependence of the two parties is also reflected in research showing an association between the life-space mobility of people with dementia and that of their family caregivers, suggesting that each person’s health and everyday activities need to be understood in relation to the other [31]. The arrangement therefore needs to be understood in relation to both the person with dementia and the family caregiver. In Sweden, municipal social care interventions are governed by the Social Services Act, according to which interventions should be designed and implemented together with the individual, take the individual’s preferences into account, and strengthen the person’s ability to live independently [4]. Respite care is intended to provide family caregivers with opportunities for rest and recovery, but its acceptability and sustainability also depend on how the person with dementia experiences the stay. Municipal decisions should consequently not be based solely on the need to relieve family caregivers or solely on the assessed needs of the person with dementia. The preferences, well-being, and circumstances of both parties need to be considered, while ensuring that the perspective of the person with dementia is not displaced by the needs or accounts of others.

This has implications for person-centered planning of respite care. Before and during an alternating housing arrangement, professionals should explore the person’s preferred routines, interests, social needs, relationship to the home, and reactions to transitions between settings. Repeated expressions of loneliness, withdrawal, or a desire to return home should prompt reassessment of the content and suitability of the care rather than being regarded as an inevitable feature of dementia. Individual adaptation may involve continuity of rooms and staff, access to familiar objects and routines, support for outdoor activity, and opportunities to participate in activities that are meaningful to the individual. It may also require considering whether another form of support, such as day services or respite care in the home, would better correspond to the person’s preferences.

The present findings further suggest that the success of alternating housing should not be judged only through group-level comparisons of quality of life or symptom scores. Individual trajectories and experiences may be more informative, particularly in small and heterogeneous populations. Future studies could therefore combine repeated assessments with interviews or observations across several respite periods to examine how experiences change over time and whether particular features of the environment, activity provision, staff continuity, or transition process are associated with more positive or negative experiences. Including both people with dementia and family caregivers while analyzing their perspectives separately could also provide a fuller understanding of how alternating housing balances the needs of the two parties.

### Strengths and Limitations

We invited all people with dementia who had been granted respite care by the included municipality at the time of data collection. Only half of them agreed to participate, giving us eight participants. The reason why several refused to participate (via family members) was due to reduced cognitive ability, meaning consent could not be obtained or that the person declined, as it was felt that an interview would be too exhausting emotionally. Of those who accepted the invitation, all except for one person were men. Although the intention was to even out the imbalance in the distribution between the sexes, the same gender distribution can also be seen in previous research in this area with the same age range as the current study. Also, as women are more often affected by dementia and men do not live as long as women, women are probably cared for in special residences instead of having respite care interventions.

From a methodological perspective, the use of CIT allowed nuanced exploration of both positive and negative experiences, providing a rich contextual understanding often missing from purely quantitative studies. Although the sample size was small (n = 8), the depth of qualitative data, combined with validated measurement instruments, increases the credibility and transferability of the findings. Moreover, the results are in line with, and extend, the previous literature in a population that remains underrepresented in research due to cognitive limitations.

The use of CIT for both data collection, i.e., interviews, and the analysis was appropriate as it allowed us to focus, in accordance with this method, on incidents, dilemmas, or events that the participants had experienced in connection with respite care. Even if the results of the current study are based on the experiences of a small number of participants, the results seem to be important as earlier research on patient group experiences of respite care is very sparse. We cannot claim to have performed measurements via validated instruments in a quantitative sense because the participants were so few. However, these measurements provide good support for what was described by the participants during the interviews. Because of the size of the sample, the quantitative component was limited to descriptive presentation and cannot be interpreted as providing statistical evidence of differences between the two housing settings. Furthermore, more recognizable patterns could have been observed over time if there had been multiple time-points. Nevertheless, the QUALID and NPI-NH assessments provided complementary information that was considered alongside the participants’ descriptions of their experiences. The study should therefore be understood as a qualitatively driven mixed-methods study in which the quantitative component had a supportive and contextualizing function.

Interviewing people with dementia requires respect, integrity, and communicative ability from the interviewer. One interview was interrupted at home, and a new attempt was made at the respite care residence but had to be interrupted there as well since it would not have been an ethically correct decision to continue due to very severe cognitive impairment. The time of day at which interviews and assessments were conducted was not systematically recorded. Consequently, possible fluctuations related to fatigue, sleep disturbance, or time-of-day effects could not be examined. The quality of life, behavioral, and psychological assessments were carried out partly with the family caregiver in ordinary housing, partly via healthcare staff at the respite care residence. In this way, there is uncertainty as the estimates were conducted by different people. On the other hand, it is a strength that the assessments were carried out with validated instruments by one and the same person in the research team. Further, using a quantitative scale may seem easy but can be exhausting for the family members, as it can be perceived as both ethically sensitive to ask them to assess their loved ones, and it can arouse strong feelings among them. The researchers in the team are also all experienced registered nurses with specialist training and used to supporting families in difficult situations. This provided a good basis for handling the data collection, even if all went smoothly.

## 5. Conclusions

Respite care appears to maintain a high quality of life and a low level of psychological symptoms for the vast majority of people with dementia. Although our sample was very small, these results are nevertheless an important contribution to a phenomenon that needs further research. Furthermore, these results can give decision-makers guidance for municipal care efforts.

Taken together, the findings indicate that alternating housing can provide stability, continuity, and opportunities for social engagement that support well-being among people with dementia. The combination of familiar routines at home and structured activities in respite settings may help sustain quality of life while also offering essential relief to family caregivers. However, to fully realize the potential of respite care, attention must be paid to individual adaptation, staff continuity, and meaningful daily activities that promote person-centered care. Future studies with larger and more diverse samples are warranted to explore how respite care can be optimized to balance the needs of both people with dementia and their family caregivers.

## Figures and Tables

**Table 1:** Characteristics of the persons with dementia (n = 8) and scores of Quality of Life in Late-Stage Dementia (QUALID) and Behavioral and Psychological Symptoms of Dementia (NPI-NH).

Item Participants		1	2	3	4	5	6	7	8	
										Mean
Age		77	80	91	84	74	80	72	87	81.6
Gender		Male	Male	Male	Male	Male	Male	Male	Female	
Marital status		Married	Married	Married	Married	Married	Married	Married	Widow	
Housing situation		Apartment	Villa	Villa	Villa	Villa	Apartment	Apartment	Villa	
QUALID, total score *										Mean
	At home	25	13	17	25	25	20	21	27	19.5
	Respite care	19	15	20	22	28	15	25	32	22.8
NPI-NH items ** Yes/No										
Delusions										
	At home	Yes	No	No	No	No	No	Yes	Yes	
	Respite care	Yes	No	No	No	No	No	No	No	
Hallucination										
	At home	No	No	No	Yes	Yes	No	Yes	Yes	
	Respite care	No	No	No	No	Yes	No	No	No	
Agitation										
	At home	Yes	No	No	Yes	No	No	Yes	Yes	
	Respite care	No	No	No	Yes	No	No	Yes	Yes	
Depression										
	At home	Yes	No	No	No	No	No	Yes	Yes	
	Respite care	Yes	No	No	Yes	Yes	No	Yes	Yes	
Anxiety										
	At home	No	Yes	No	Yes	No	Yes	Yes	Yes	
	Respite care	No	Yes	No	Yes	Yes	No	Yes	No	
Excitement/Euphoria										
	At home	No	No	No	No	No	No	No	No	
	Respite care	No	No	No	No	No	Yes	No	No	
Apathy/Indifference										
	At home	Yes	No	Yes	Yes	Yes	No	No	Yes	
	Respite care	No	Yes	No	Yes	No	No	No	No	
Incontinence										
	At home	No	No	No	Yes	No	No	Yes	No	
	Respite care	No	No	No	No	No	No	No	Yes	
Ease/Lability										
	At home	No	No	No	Yes	No	No	Yes	No	
	Respite care	No	No	No	Yes	No	No	No	Yes	
Motoric restlessness										
	At home	Yes	No	No	No	Yes	Yes	Yes	No	
	Respite care	No	Yes	No	Yes	Yes	No	Yes	No	
Sleep disorders										
	At home	Yes	No	No	No	Yes	Yes	Yes	No	
	Respite care	No	Yes	***	***	Yes	No	Yes	No	
Appetite/eating disorders										
	At home	No	No	No	No	No	Yes	Yes	No	
	Respite care	No	No	No	No	No	No	Yes	No	

NOTE: * Quality of life in late-stage dementia, 11 points = highest degree of quality of life, 55 points = lowest degree of quality of life; ** Neuropsychiatric Inventory scale—Nursing home, items for screening YES/NO; *** missing.

## Data Availability

The datasets generated and/or analyzed during the current study are not publicly available due to Swedish regulations and laws but are available from the corresponding author upon reasonable request.
